# Excess cerebral TNF causing glutamate excitotoxicity rationalizes treatment of neurodegenerative diseases and neurogenic pain by anti-TNF agents

**DOI:** 10.1186/s12974-016-0708-2

**Published:** 2016-09-05

**Authors:** Ian A. Clark, Bryce Vissel

**Affiliations:** 1 Biomedical Sciences and Biochemistry, Research School of Biology, Australian National University, Acton, Canberra, Australian Capital Territory 0200 Australia; 2 Neurodegeneration Research Group, Garvan Institute, 384 Victoria Street, Sydney, New South Wales 2010 Australia

**Keywords:** TNF, Glutamate, Astrocyte, Synapse, Glutaminase, Re-entry proteins, Neurodegenerative disease, Neurogenic pain

## Abstract

The basic mechanism of the major neurodegenerative diseases, including neurogenic pain, needs to be agreed upon before rational treatments can be determined, but this knowledge is still in a state of flux. Most have agreed for decades that these disease states, both infectious and non-infectious, share arguments incriminating excitotoxicity induced by excessive extracellular cerebral glutamate. Excess cerebral levels of tumor necrosis factor (TNF) are also documented in the same group of disease states. However, no agreement exists on overarching mechanism for the harmful effects of excess TNF, nor, indeed how extracellular cerebral glutamate reaches toxic levels in these conditions. Here, we link the two, collecting and arguing the evidence that, across the range of neurodegenerative diseases, excessive TNF harms the central nervous system largely through causing extracellular glutamate to accumulate to levels high enough to inhibit synaptic activity or kill neurons and therefore their associated synapses as well. TNF can be predicted from the broader literature to cause this glutamate accumulation not only by increasing glutamate production by enhancing glutaminase, but in addition simultaneously reducing glutamate clearance by inhibiting re-uptake proteins. We also discuss the effects of a TNF receptor biological fusion protein (etanercept) and the indirect anti-TNF agents dithio-thalidomides, nilotinab, and cannabinoids on these neurological conditions. The therapeutic effects of 6-diazo-5-oxo-norleucine, ceptriaxone, and riluzole, agents unrelated to TNF but which either inhibit glutaminase or enhance re-uptake proteins, but do not do both, as would anti-TNF agents, are also discussed in this context. By pointing to excess extracellular glutamate as the target, these arguments greatly strengthen the case, put now for many years, to test appropriately delivered ant-TNF agents to treat neurodegenerative diseases in randomly controlled trials.

## Background

The amyloid theory of Alzheimer’s disease, and by extension other chronic neurodegenerative states, has dominated the field for decades. It has, however, in the face of the reality of numerous large clinical trials yielding no clinical improvement, lost momentum. A recent item on the Editor’s Blog of the webpage of the *Journal of Alzheimer’s Disease* entitled “Time to Dismount” (see http://www.j-alz.com/editors-blog/posts/time-dismount) eloquently again brings to the fore the long-held, widespread, and increasing unease among researchers [1–5]. Likewise, outcomes comparing in vivo human cerebral amyloid β (Aβ) deposition on Pittsburgh Compound B PET imaging have not generated optimism for the amyloid theory [6, 7]. Recent key epidemiological evidence from a large population in which administering regular subcutaneous etanercept over an extended period in treatment of rheumatoid arthritis (RA) patients was reported to reduce incidence of Alzheimer’s disease (AD) [8], further reduces the likelihood of Aβ being the key to AD pathogenesis.

We have recently [9] reviewed the literature demonstrating that increased soluble Aβ does not cause direct damage but is one of the proinflammatory cytokine-induced damage-associated molecular patterns (DAMPs) recognized by toll-like receptors (TLRs). These receptors also recognize pathogen-associated molecular patterns (PAMPs) present on the surface of, for example, the microbes now widely agreed to be sometimes associated with AD [10]. Agonists of TLRs, which are on and in various types of cells, including those throughout the brain, release more of these same cytokines, including tumor necrosis factor (TNF). This is consistent with Aβ not inhibiting long-term potentiation in hippocampal slices from mice treated with anti-TNF agents, such as infliximab [11]. Clearly, from the literature we have recently quoted [9], Aβ is best regarded, along with S100 proteins and high-mobility group box 1 (HMGB1), as belonging to a class of DAMPs (secondary DAMPs) that exacerbates production of the proinflammatory cytokines responsible for their own increase, and induces them further, causing a forward feed chain reaction. Moreover, variation in levels of these other DAMPs of this same class, possessing the same TLR-mediated, TNF-generating activity in AD, may explain why normal aged patients can exhibit high Aβ plaque levels. It may also explain why removing soluble Aβ or its plaque, still the goal of the many clinical trials [12], does not retard human disease progression, since the other secondary DAMPs, S100 proteins and HMGB1, are still actively inducing TNF. In contrast, removing Aβ is successful in mouse transgenic models that have been designed to generate pathologically but artificially high Aβ [13].

Waning enthusiasm for the amyloid theory now allows many other approaches, including the last 10 years of animal studies, case reports, open trials, and off-label treatments of neurodegenerative diseases, based on neutralizing excessive levels of TNF within the brain, to receive more attention. Unaccountably, this neglected approach to neurodegenerative disease is sometimes still referred to as highly controversial [14]. This review provides the logic for increased extracellular cerebral glutamate being the central mechanism by which excessive TNF harms cerebral function and structure. TNF is the first endogenous mediator to be documented as simultaneously influencing extracellular cerebral levels of extracellular glutamate by both enhancing its release and reducing its re-uptake. Given the broad ramifications of glutamate-induced excitotoxicity in infectious and non-infectious disease, these additional layers of information about TNF provide insights with widespread therapeutic implications. In particular, it increasingly rationalizes accounts of the usefulness of neutralizing excess cerebral levels of TNF in neurodegenerative disease.

As well as providing sufficient background to enable the bigger picture of TNF in brain disease pathogenesis to be understood, we focus here on the implications of newer data, largely neglected in the world of neurodegenerative disease, on how this cytokine evidently controls levels of extracellular glutamate in the synaptic cleft. In brief, glutamate is the chief physiological excitatory neurotransmitter, essential of course in memory and learning, and indeed is functionally involved in virtually all activities of the nervous system. Glutamate’s combination of functional importance and toxicity demands tight control over its release and re-uptake. Thus, as will be discussed, control by TNF of both of these functions gives treatments based on reducing excess cerebral levels of this cytokine a solid therapeutic foundation in neurodegenerative disease, in part because of its essential effects in driving excitotoxicity. In practice, we may usefully view TNF toxicity and glutamate toxicity as two perspectives on the one pathophysiological entity.

## TNF, an extremely pleiotropic cytokine

TNF was recognized, and named, as an endogenous tumor killing agent [15], and 6 years later, its wider biological importance began to be appreciated through its roles in innate immunity and the pathogenesis of infectious disease ([16], reviewed in 2004 [17]). In due course, fundamental roles for this cytokine in physiological homeostasis [18] and non-infectious disease [19] began to be explored. After being recognized as an early step in the inflammatory cytokine cascade [20], TNF began to achieve its present wide acceptance as a master cytokine in disease pathogenesis through infliximab, the first of the specific neutralizing biological anti-TNF agents, becoming a striking clinical success in treating RA [21]. Others from this research group showed that TNF is a master cytokine through observing that infliximab reduces levels of other inflammatory cytokines as well as TNF [22, 23].

The extraordinarily broad relevance of TNF in biology can now be inferred by its strongly conserved state, traceable back through a remarkably ancient lineage including fish and insects, with the form generated by reef-building corals, and the TNF receptors on their cells, co-recognizing their human counterparts [24]. Unsurprisingly, therefore, every organ, including the brain, has proved to be influenced by this cytokine. By 1987, TNF had been shown to be a necessary part of the chain of events that control normal sleep [25], and a few years later, current conductance in neurons of a sea slug, *Aplysia kurodai*, was observed to be reduced by human TNF [26, 27]. Next, physiological levels of TNF had been reasoned to be necessary for normal mammalian neuronal function, with a loss or gain of TNF beyond homeostatic limits being pathological [28]. Nevertheless, data on other proinflammatory or anti-inflammatory cytokines such as IL-1β, IL-4, IL-17, and IL-23 [29–31] continue to add to the principles behind this concept and may well generate related therapeutic avenues.

## TNF excess in neurodegenerative states

Twenty years ago, the involvement of unchecked chronic TNF generation, particularly within the brain, in the pathogenesis of stroke, traumatic brain injury (TBI), and AD began to be apparent [32–34]. Refinements of these scientific arguments have accumulated to the present day [35–41]. The subtle relationship between these cytokines and the brain has been nicely put by noting that even when it appears that the nervous system is succumbing to a flared immune system, and the two systems maintain a constant dialogue in the attempt to restore homeostasis [42].

The rationale for treating chronic neurodegenerative states by reducing excess cerebral TNF extends far beyond the post-stroke syndrome, AD, and TBI noted above. Despite “belonging” to various disciplines, these cerebral states characterized by TNF excess clearly have much pathophysiology in common. They include (Table 1) Parkinson’s disease (PD) [43], neurogenic pain [44–50], Huntington’s disease [51], amyotropic lateral sclerosis [52], septic encephalopathy [53], defective post-operative cognition [54, 55], defective post-irradiation [56] and post-chemotherapy [57, 58] cognition, defective cognition during RA [48], epileptic seizures [59, 60], viral encephalitides [61], cerebral malaria [62], and HIV dementia [63]. Moreover, recent evidence has very precisely incriminated excess brain TNF in the pathogenesis of AD [64]. The authors employed a novel multivariate regression modeling approach, termed partial least squares regression, to investigate cytokine protein concentrations in brain tissue from AD and control patients. Taking into account the order in which brain regions are known to be impacted during the development of AD, region-specific profiles were used to identify high concentrations of cytokines which, when used alone, killed neurons in vitro. Of the 48 cytokines monitored, only TNF (=TNFα in their text) met this condition. This is entirely consistent with the evidence we have previously presented [37] that increased cerebral TNF is the most logical therapeutic target for countering this disease. As we review here, the largely neglected evidence that variation in TNF, through regulating both the release and clearance of cerebral glutamate, seems destined to widen an appreciation of this cytokine within neuroscience as a mediator of plasticity and excitotoxicity.

**Table 1:** Association of excess TNF and glutamate in brain in neurodegenerative states. See text for references

Disease	Excess brain TNF	Excess brain glutamate
Alzheimer’s disease	+	+
Parkinson’s disease	+	+
Huntington’s disease	+	+
Amyotropic lateral sclerosis	+	+
Septic encephalopathy	+	+
Traumatic brain injury	+	+
Stroke	+	+
Poor post-operative cognition	+	+
Poor post-irradiation cognition	+	+
Poor post-chemotherapy cognition	+	?
Poor cognition in rheumatoid arthritis	+	?
Epileptic seizures	+	+
HIV dementia	+	+
Cerebral malaria	+	+
Neurogenic pain	+	+
Viral encephalitides	+	+

## Glutamate in brain physiology and pathophysiology

l-glutamate, the most abundant extracellular amino acid in the brain, is, as reviewed over the decades [65–67], the chief physiological excitatory neurotransmitter, including in normal memory and learning. Cerebral glutamate is formed, in microglia and astrocytes [68], as well as neurons, by glutaminase acting on glutamine, and becomes extracellular. Homeostasis is normally maintained by a balance between this reaction and glutamate re-uptake from the synaptic cleft by a series of transport, or re-uptake, proteins that initiate its recycling back to glutamine. As discussed below, much literature associates TNF with glutamate regulation. Both too much or too little, TNF and glutamate are harmful. In brief, a plausible paired physiological role for them is low fluctuating levels of TNF determining physiological levels of glutamate in hippocampal homeostatic synaptic plasticity [69, 70], as described below.

As reviewed [66, 71], should extracellular cerebral glutamate become excessive, whether through excess release or poor clearance, or both, a harmful excitotoxicity ensues. From the 1990s, understanding the disruptions that can cause this increase has been an intense focus of interest in the pathophysiology of neurodegenerative diseases. These conditions (Table 1) came to include AD [72], PD [73], Huntington’s disease [74], amyotropic lateral sclerosis [75], stroke [76], viral encephelitides [77, 78], septic encephalopathy [79], defective post-operative cognition [80], post-irradiation brain function [81], pain [82, 83], bacterial meningitis [84], epileptic seizures [85], human immunodeficiency virus (HIV) dementia [86], cerebral malaria [87], and TBI [88–90]. In addition, the key studies of Jourdain and co-workers [91] convincingly combined functional and ultrastructural evidence to argue the case for glutamate from astrocytes being a key player in physiological control of synaptic strength. Increasingly, these glutamate pathways have therefore become essential background reading for those whose chief interest has been developing therapeutic drugs for treating these conditions. A recent comprehensive review [92] provides a clear account of the complexities of the control of cerebral extracellular glutamate in chronic, as distinct from acute, excitotoxicity in neurodegenerative states, and discusses amyotrophic lateral sclerosis (ALS), AD, and Huntington’s disease as examples. However, this text takes no account of the presence of excess cerebral TNF production or its influence on extracellular brain glutamate levels in these and similar diseases [34, 51, 93–95].

## The roles of excess cerebral TNF in generating glutamate toxicity

### Inhibition of re-uptake proteins

As reviewed in 2001 [96], glutamate re-uptake from the synaptic cleft noted above is controlled by fluctuations in a unique family of amino acid transport, or re-uptake, proteins that act as signal terminators. Their inhibition is intricately involved in the pathogenesis of glutamate-excess excitotoxicity diseases such as stroke, AD, epilepsy, and chronic pain syndromes. Twenty years ago, TNF was first implicated in generating excitotoxicity through its capacity to inhibit glutamate re-uptake in an HIV dementia model [97] and subsequently in cultures brain slices [98] and a Sindbis virus disease model [99]. Although outside the topic of this review, which discusses entry of glutamate into the synaptic cleft rather than its actions while there, we note that emphasis has more recently been placed on the ability of TNF to regulate the various types of glutamate receptors [100]. The details of control of these transport proteins by TNF have more recently been updated in a rat model of ALS [101]. When combined, the ideas generated in these fields of research have allowed insightful functional links of neuroinflammation and glutamate-induced excitotoxicity to be proposed [102, 103].

## Glutaminase upregulation

**Fig. 1:**
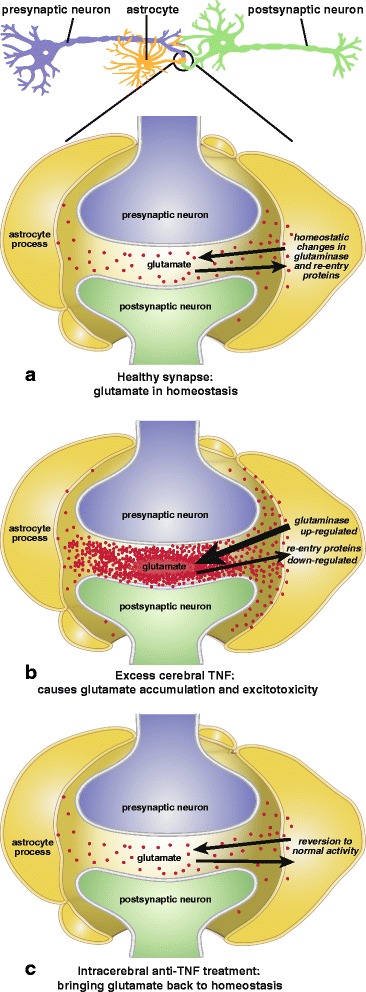
**a** Normal synapse, with physiological variations in TNF controlling glutamate levels in synaptic cleft through homeostatic activity of glutaminase and re-entry transporter proteins. **b** Excess cerebral TNF enhancing glutaminase and inhibiting re-entry transporter proteins, causing glutamate to accumulate to excitotoxic toxic levels. **c** Glutamate excess rapidly dispersed from synaptic cleft due to glutaminase reduction plus re-entry protein upregulation. Both occur together after treatment with intracerebral (perispinal) anti-TNF biologicals or non-specific TNF inhibitors (dithio-thalidomines, nilotinib, cannabinoids) by other routes. Glutaminase reduction alone occurs with DON, and re-entry protein upregulation alone with ceftriaxone and riluzole

In the event, TNF became much more heavily incriminated in glutamate regulation than has been taken into consideration in the above models of excitotoxocity (Fig. 1). Ten years ago, this cytokine was reported to generate excessive glutamate levels by markedly upregulating glutaminase activity [104]. This was confirmed, as was a concomitant reduction in glutamate re-uptake, in a model of Japanese viral encephalitis [105]. The next year, with the same surprisingly little influence on mainline excitotoxicity research to date, glutaminase upregulation was reported after stimulating primary cultured human neurons with TNF or interleukin-1β [106]. Furthermore, these authors found the glutamate increase to occur in the extracellular space as well as intracellularly. The following year, this group also showed that etanercept reduces inflammation and lethality in the above model of Japanese viral encephalopathy [107]. Clearly, by increasing glutamate production while simultaneously reducing its re-uptake [97], excess TNF can be expected to readily cause glutamate to accumulate to toxic levels. This implies much more therapeutic potential for anti-TNF agents than other drugs possessing only one of these activities, such as 6-diazo-5-oxo-norleucine (DON), ceftriaxone, or riluzole, as discussed below. However, this TNF-glutaminase link, despite first being made a decade ago (above), does not yet appear to be common currency in neurodegenerative disease circles (e.g., [108]). Readers interested in the complexities of glutamate release, including its physiological control, are directed to the examples provided by references [109–111].

In passing, we note that glutamine deficiency is a long-recognized characteristic of chronic inflammatory stress and has made nutritionally motivated i.v. glutamine replacement therapy a routine, if formally untested, adjunct treatment in critical care wards [112]. However, a recent post hoc analysis of a large-scale randomized trial has shown this procedure to be of no value, perhaps even harmful [113]. This raises the possibility that chronic TNF increase present in these patients may have caused the observed glutamine depletion by the combined effects of enhancing its conversion to glutamate, plus inhibiting its reconversion from glutamate, as summarized above. Clearly, amino acids have many functions as well as providing nutrients.

## Glutamate toxicity as a major manifestation of excess TNF in brain disease

The above data on TNF provide insights into the breadth of therapeutic relevance of the functional link between unbridled TNF production and glutamate neurotoxicity and how this adds immensely to the central argument of this review that TNF is a highly logical target in neurodegenerate disease. As but one example, the capacity of excess TNF to greatly increase glutamate output through activating glutaminase [104, 105] casts the considerable body of work on astrocytes, glutamate, and basal ganglia excitotoxicity, in which the influence of inflammatory cytokines are not considered [114], in a new light. It also modifies the novel “glutamate grabber” approach to treating brain ischemia [115], in that etanercept is, from the above insights, likely to be much more effective than glutamate-oxaloacetate transamimase or oxaloacetate by preventing an excess of newly formed glutamate.

Importantly, intra-amygdala infusion of TNF has been reported to elevate glutamate levels in this region of the brain [116]. Likewise, etanercept, a specific anti-TNF biological in wide clinical use, lowers brain glutamate levels in experimental models (Table 2). Although etanercept is too large a molecule for all but a small amount of a subcutaneous (s.c.) dose to enter the cerebrospinal fluid (CSF), intentionally compensating for this by giving a 20-fold larger dose reduces brain glutamate in a rat model of traumatic brain injury [117]. Etanercept has also been reported, in a heart failure model in which TNF is increased [118, 119], to lower rat brain glutamate dramatically when given intracerebroventricularly (i.c.v), although, again because of its high molecular weight, not when administered intraperitoneally (i.p.).

**Table 2:** Outcome of administering specific or non-specific anti-TNF agents in states exhibiting excess cerebral TNF and the opposing effects of TNF and anti-TNF agents of brain glutamate levels. See text for references

	Excess cerebral TNF present	Positive outcome after etanercept, etc.	Positive non-specific TNF inhibitors outcome		
			Thalid or dithio-thalid	Nilotinib	Cannabinoids
Alzheimer’s disease	+	+	+	+	+
Parkinson’s disease	+	?	?	+	+
Huntington’s disease	+	?	?	?	?
Amyotropic lateral sclerosis	+	+	?	?	?
Septic encephalopathy	+	?	?	?	?
Traumatic brain injury	+	+	+	?	+
Stroke	+	+	+	?	?
Poor post-operative cognition	+	+	?	?	?
Poor post-chemother cognition	+	?	?	?	?
Poor post-irradiation cognition	+	?	?	?	?
Epileptic seizures	+	?	?	?	+
HIV dementia	+	?	?	?	+
Neurogenic pain	+	+	?	?	+
Viral encephalitides	+	?	?	?	+
Elevated brain glutamate	+				
Lower brain glutamate		+	?	?	+

## Do these actions of TNF explain the rapid response to etanercept in neurodegenerative disease?

Control of glutamate by TNF might also explain why etanercept has often been reported to reverse a number of clinical manifestations of neurodegenerative disease surprisingly rapidly. It was shown 17 years ago [120] that turnover of cerebral extracellular glutamate is very fast, seconds to minutes in these authors’ hands. This is evolutionarily essential because of the key role of this amino acid in the synaptic cleft, where it is responsible for the fast excitatory neurotransmission necessary for the rapid brain responses demanded for survival in the real world. Thus, given the role of TNF to influence both glutaminase and re-uptake proteins described earlier, the capacity of intracerebral etanercept to lower brain glutamate, as summarized above [117–119], can be expected to act with somewhat the same degree of rapidity. It seems likely, therefore, that these data rationalize the unexpected but clearly rapid response in case reports and open trials to perispinal etanercept, initially reported in 2003 [121] and 2008 [122], and regularly confirmed since [123–127]. Awareness of this 1999 report on the rapidity of extracellular cerebral glutamate turnover [120] may now help contribute to the body of accruing evidence that should alter attitudes regarding reports of rapid responses to anti-TNF in neurodegenerative disease [126, 128].

## Therapeutic implications for excitotoxicity in neurodegeneration

### Specific anti-TNF biologicals

Whereas infliximab and adalimumab are essentially monoclonal antibodies directed at TNF itself, etanercept, the only anti-TNF biological drug yet tested in this context, is a fusion protein consisting of the p75 TNF receptor, joined to the constant end of the IgG1 antibody [129]. An etanercept biosimilar is already in the literature [130, 131], and a number of others already have approval or are being developed [132], providing the prospect of reduced treatment costs for most of the world. Recent debates on competitive pressures versus scientific rationales delaying introduction of biological biosimilars are most informative [133]. Clearly, regulating this field is a state of flux.

The use of anti-TNF agents in neurodegenerative disease has its critics who largely base their concerns on whether the functional complexities of TNF science, such as the p55 and p75 TNF receptors and membrane versus soluble location of TNF, should be more fully elucidated beforehand [134, 135]. This is largely overplayed: clinical development of the specific anti-TNF biologicals in RA, psoriasis, and Crohn’s disease went ahead successfully during the past decades alongside continuing yet incomplete basic research without such reservations being aired. The case for cautious use of specific biological anti-TNF agents based on the soundness of the pathophysiological arguments in otherwise untreatable conditions, with an eye to potential concerns, has been amply made in systemic states [136]. In practice, the balance of safety versus outcomes has proved to be very much on the side of the millions of patients who have received regular treatment with these agents for many years now.

## Blood-brain barrier (BBB) passage by specific anti-TNF biologicals

As other therapeutic molecules, the biological anti-TNF agent etanercept, though employed widely with great success systemically, is, as often noted, too large to cross the BBB in significant amounts unaided. Two BBB-crossing techniques now exist side by side in the literature, the earliest and simplest from a small group, the later technically complex, and not yet tried in patients. Here, we summarize their origins and rationale.

### Perispinal delivery of etanercept

Understanding the perispinal delivery of large molecular weight drugs into the central nervous system requires an appreciation of the cerebrospinal venous system. As recently reviewed in detail [137], this route of cerebral venous drainage has had, since its discovery well over a century and a half ago, a most complex and interesting history, and more recently application, in medical advances. Contemporary awareness of the potential of this route began when researchers in aviation medicine were exploring an animal model of the effects of gravity and body position on pilots of high-performance aircraft [138]. They noted that restraining anesthetized rabbits on a tilt board and rotating them to a head-down position considerably increased CSF levels of the plasma protein albumin within 5 min. The authors noted, in passing, that as well as aiding their branch of science, their data had implications for getting large molecular weight therapeutics into the brain. As discussed [139, 140], the principle behind this approach—drug delivery to the brain by retrograde venous flow—began to be used off-label the early 2000s to get etanercept into the brain in patients with neurogenic pain [121] and AD [141]. Although these open trial observations (Table 2) continue to be reported post-stroke [125] and TBI [125], and the principles they embrace now have a solid foundation in animal models [50, 55, 142–144], remarkably they have not, as did parallels in inflammatory states in other organs earlier, attracted funding for randomized human trials. As noted above, a wider awareness of the rapid rate of glutamine to glutamate kinetics [120] may well, through rationalizing the reported rapid response [121, 122], reduce skepticism.

### Trojan Horse delivery of etanercept

An alternative method of delivery of large molecules into the brain exists [145–147], but it ignores the above input from aviation medicine and, as has been discussed [148–150], remains fraught with technical difficulties. In 2011, the UCLA/Armagen group reported that a re-engineered version of etanercept, in which the IgG part of the fusion protein is a chimeric monoclonal antibody against the mouse transferrin receptor, could be delivered into the mouse brain in this way [151]. The following year, they reported that this re-engineered etanercept reduced the harmful effects of experimental stroke in a mouse model [152]. It cannot, however, be tested in humans until etanercept is re-engineered so that it recognized human, as distinct from mouse, transferrin. Moreover, re-engineering is essential for each large molecule under consideration, whereas they can be expected to function in their original form when introduced perispinally. A recent review [153] discusses a number of further complexities that need addressing before Trojan Horse delivery could become routinely used.

## Non-specific inhibitors of TNF

### 3,6 Dithio-thalidomides

Thirty years after being removed from the market in 1961 because of its disastrous effects on fetal development, thalidomide had begun to be explored to treat a number of intractable conditions in patients other than child-bearing age women. It was shown to selectively inhibit TNF production by stimulated human monocytes [154], and to do so by enhancing degradation of the mRNA for this cytokine [155]. A decade later, a series of thio-thalidomides with higher anti-TNF effects than the parent compound were synthesized [156], and the outcomes of their use on TNF mRNA generation closely compared [157]. A considerable literature now exists on some compounds of this class, which are orally active, pass the blood-brain barrier (BBB) and improve outcome in neurodegenerative disease models by inhibiting TNF. They are widely efficacious, by various behavioral and cognitive criteria, in models of lipopolysaccharide-induced neuroinflammation [158], AD [159–161], TBI [55], and stroke [142]. Recently, the parent compound has been reported to reduce a form of neurogenic pain by repressing the inflammatory response [162]. We are unaware of any literature on dithio-thalidomides influencing glutaminase, but the parent compound also has been reported to prevent hypoxia-induced TNF from inhibiting one of the glutamate re-uptake proteins [163].

### Nilotinib

Nilotinib, a tyrosine kinase inhibitor, is 30 times more potent than imatinib, which it is replacing for treating certain leukemias [164, 165]. A small open trial of 6-month duration performed at Georgetown University Hospital was reported at the most recent Annual Meeting of the American Society for Neuroscience. Daily oral nilotinib showed promise, in an initial uncontrolled trial, of reversing clinical aspects of PD with or without dementia, as well Lewy Body dementia [166]. Phosphorylated tau (P-tau), α-synuclein, and Aβ were noted to have been significantly reduced [166] in nilotinib-treated patients. Previously, nilotinib had been reported, by this group and others, to be successful in controlled studies in mouse PD models [167–169]. One of these groups [169] demonstrated that a clinically useful proportion of orally administered nilotinib, as used in this new open trial [166], passes through the BBB. Prior animal studies also show that using nilotinib and the closely related dasatinib was useful in models of AD [170–172].

The capacity of nilotinib (and indeed dasatinib [170]) to inhibit TNF generation in vivo [173, 174], and the observations that blocking TNF duplicates this effect of nilotinib in PD models [43, 175], appear to have not yet been considered as a plausible mechanism of these new clinical observations with this agent [166]. Nevertheless, as an anti-TNF agent, nilotinab can be expected, from the activity of TNF in these contexts [97, 104], to inhibit glutaminase activity as well as enhance glutaminate re-uptake proteins. In addition to nilotinib [176] another selective Src tyrosine kinase, pyrazolopyrimidine-2 (PP-2), inhibits production of TNF [177]. Also, given that insulin resistance is a common direct consequence of chronically increased TNF [178], further evidence for anti-TNF effects being central to these observations with nilotinib comes from the ability of these tyrosine kinase inhibitors to treat type 2 diabetes mellitus (T2DM) by decreasing insulin resistance [179].

How might nilotinib reduce TNF levels? Tyrosine phosphorylation is central to TLR stimulation and subsequent activation of NF-kappaB [180] that generates cytokines such as TNF. Endotoxin tolerance is associated with inhibited phosphorylation Src, a non-receptor tyrosine kinase protein [181]. It is therefore plausible that agents such as nilotinib, which inhibit Srcs, reduce TNF production [173, 174] by mimicking tolerance to TLR agonists such as endotoxin.

## Cannabinoids

As reviewed [182], the therapeutic and pharmacological secrets of *Cannabis sativa* have fascinated researchers for about two centuries. About 90 phytocannabinoids (i.e., compounds present in the plant) have been identified, the two with the largest literatures being tetrahydrocannabinol (THC) and cannabidiol (CBD). The former is psychotropic and thus under a legal cloud, although a synthetic trans-9-delta isomer, termed dronabinol, is an example of forms of THC nowadays undergoing limited investigation [183]. CBD, in contrast, does not cause significant behavioral change and is researched much more widely. These phytocannabinoids, self-evidently BBB permeable, proved to be ligands for two previously unsuspected receptors, mainly found on cells of the immune system, and whose presence led to the prediction and discovery of endogenous cannabinoids, or endocannabinoids. In physiological terms, these may be considered as part neurotransmitter, part cytokine, and part hormone and have been identified and studied at length (see [184, 185] for reviews.)

Both endocannabinoids and CBD have been shown to be active in models for pain [186–190], AD [191–195], epileptic seizures [196–199], PD [200–202], HIV dementia [203–205], viral encephalitis [206], and TBI [207]. Clearly, this list parallels the conditions, discussed earlier, with which excessive cerebral levels of TNF are associated. These agents are also active in hypoxic encephalopathy, a stroke parallel in newborns [208, 209], a condition associated with raised inflammatory cytokines and glutamate [210]. Not surprisingly, therefore, cannabinoids, whether synthetic, endogenous, or of plant origin, have proven to be established anti-TNF agents in vitro and in vivo, in the sense that they reduce its production by the usual recognized stimuli [211–214]. This list includes treating the murine malarial encephalopathy (cerebral malaria) [214], a condition in which, as discussed below, 6-diazo-5-oxo-norleucine is also efficacious for a related and predictable reason concerned with lowering extracellular cerebral glutamate [215].

Again, the list of model conditions investigated for therapeutic use of cannabinoids in the previous paragraph remarkably mirrors the list of previously discussed conditions associated with excessive cerebral extracellular levels of glutamate. Moreover, treatment with cannabinoids or altering the function of their cellular receptors [185, 191, 216–221] has been reported to lower the levels, or function, of brain glutamate (Table 2). This is entirely consistent with their activity as anti-TNF agents [211–214].

## Agents that do not influence TNF but still reduce extracellular brain glutamate

### 6-Diazo-5-oxo-norleucine (DON), a glutaminase inhibitor

DON, a glutamine analogue, is studied largely with a view to reduce extracellular glutamate, and thus treat glutamate toxicity, through inhibiting glutaminase. Having been earlier shown [222] to possess anti-tumor properties, nearly 40 years later, DON was reported to inhibit glutaminase and thus reduce the release of glutamate in the rat cerebral cortex [223]. Through the last decade, DON has been a useful, albeit often toxic [224], experimental tool to demonstrate that glutamate-mediated excitotoxicity is a significant component of the pathogenesis of various neurodegenerative states, including brain ischemia [225]. Cerebral glutamate homeostasis is disrupted in mouse models of both the neurological sequelae of Sindbis virus infection [226] and malarial encephalopathy caused by *Plasmodium berghei* ANKA [87], and DON has been successfully used therapeutically in experimental versions of both conditions [215, 227]. It has also been useful in an in vitro HIV dementia model [228] and in both in vitro and ex vivo experimental autoimmune encephalitis, a mouse model of multiple sclerosis [229].

These new data on DON and the relationship between TNF and glutamate excess through glutaminase enhancement put historic observations on malarial encephalopathy into clearer focus. As has been reviewed [17], malaria was the first disease, infectious or otherwise, for which TNF was argued to be central to its pathogenesis, and it set the pattern for the rest. Yet a large trial of a specific anti-TNF antibody, injected intravenously, failed to show evidence, in a large trial in West Africa, of any protective effect in children with cerebral malaria [230]. At that time, however, ideas on malarial disease were predicated on harmful levels of TNF being produced intravascularly, where the parasites that stimulate its production reside, so it was considered logical to administer the antibody into this compartment. Not until 8 years ago was the excess TNF in cerebral malaria shown to originate in the brain [62]. These authors predicted that interventions to decrease TNF production in the brain might be required in order to improve outcomes. Thus, treating human cerebral malaria with perispinal etanercept, evidently an equivalent to administering it i.c.v. [125], will have at least as good and theoretically better ability—since it would also enhance re-uptake proteins—as DON to reduce excessive levels of glutamate, and therefore improving clinical outcome. DON, a molecule not known to affect re-uptake proteins, but which inhibits glutaminase [223], as well as passes the BBB when given i.p. [231], has recently also been effective in treating mice infected with Sindbis virus [227] as well as the cerebral malaria model discussed above [215].

### Ceftriaxone and riluzole, glutamate re-uptake transporter enhancers

Ceftriaxone is a broad-spectrum beta-lactam antibiotic, largely reserved, in this context, for use against otherwise resistant bacteria. In contrast to cannabinoids and nilotinib, it has been shown not to reduce TNF release from LPS-treated human monocytes [232], implying it does not act against excitotoxicity by inhibiting TNF-mediated glutaminase enhancement [97] or enhancing TNF-mediated reduced glutamate re-uptake [104]. In 2007, ceftriaxone was shown to independently enhance glutamate re-uptake and thus reduce the glutamate-dependent portion of morphine-dependent hyperthermia [233]. This activity of ceftriaxone was soon shown, in primary fetal human astrocytes, to operate through increased expression of excitatory amino acid transporter 2 (EAAT2) promoter activity, allowing it to inhibit glutamate-induced excitotoxicity of its own accord [234]. As these authors noted, this implies that ceftriaxone could have therapeutic activity in a range of neurodegenerative conditions, essentially the examples we discussed earlier as exhibiting excitotoxicity. With this mechanism in mind, ceftriaxone is nowadays under active consideration as a therapy in models of AD [235], stroke [236], TBI [237–239], and PD [240–243]. The most complete evidence consistent with this approach to date is a very recent extensive report on ceftriaxone rescuing brain function in *Toxoplasma gondii*-infected mice [244]. The authors documented high brain glutamate, although how this arose remains uncertain. *T. gondii* is a well-known TNF inducer. Being a pathogen, it possesses the PAMP activity discussed earlier.

Riluzole (6-(trifluoromethoxy)benzothiazol-2-amine), a relatively toxic material nevertheless approved for treatment of ALS, has for some time been known to be a glutamate release inhibitor and thus affecting the glutamate functions discussed above. This has been reported to include enhancing levels of glutamate re-uptake transporters [245], including in astrocytes [246, 247]. This principle is entirely consistent with findings in a mouse AD model [248] and has been extended in a recent study in which riluzole proved to reverse the same array of human gene changes in AD and aging [249]. Both research groups suggest the effects of riluzole as a possible mechanism underlying its improvement in cognitive function in their studies.

## Relative effectiveness of these treatments

As discussed earlier, neurodegenerative diseases are characterized by excessive levels of extracellular cerebral glutamate that can be expected to have accumulated through its too rapid formation as well as its slowed re-uptake and conversion back to glutamine. The ideal therapy would be able to reverse both of these changes. So far as we are aware, TNF is the only endogenous mediator that, when in excess, as in the brain in these diseases, enhances cerebral glutaminase [104] and also inhibits glutamate re-uptake proteins [97]. Since anti-TNF agents, both specific and non-specific, can be predicted to simultaneously reverse both of these TNF-induced changes, their efficacy in reversing this excitotoxicity can be expected, from first principles, to be higher than agent such as DON, ceftriaxone, or riluzole, which correct only one of these two pathways.

Specific anti-TNF biologicals are expensive, but can be very effective through neutralizing a precise target, in this case excessive cerebral TNF, known to be central to the disease in question. As discussed earlier, their large size need not be a problem. In contrast, pharmaceuticals such as dithio-thalidomides, nilotinib, cannabinoids, DON, ceftriaxone, and riluzole, although less expensive, may prove to be burdened with unknown targets that generate greater side effects than anti-TNF biologicals can. They have, however, a considerable advantage in neurodegenerative disease in that when administered orally or systemically, they can traverse the BBB and get to where they are needed [143, 157, 169, 231].

## Conclusions

We propose that the excess levels of TNF, and glutamate in the brain across a range of neurodegenerative diseases are crucially linked, high TNF causing extracellular glutamate to accumulate to levels high enough to inhibit synaptic activity and kill neurons by two synergistic mechanisms. As described, these are increasing glutamate production by enhancing glutaminase and simultaneously reducing glutamate clearance by inhibiting re-uptake proteins, thus causing it to accumulate in the synaptic cleft. The shared efficacy of specific anti-TNF biologicals and non-specific anti-TNF agents (thio-thalidomides, nilotinib and cannabinoids) on this superficially diverse range of conditions can thus be understood. The usefulness of DON, ceftriaxone, and riluzole, agents without apparent anti-TNF activity, but each possessing separate activities that counter one of these two influences of high TNF on glutamate accumulation, are similarly rationalized.

## Abbreviations

Aβ: Amyloid-β
ALS: Amyotrophic lateral sclerosis
AD: Alzheimer’s disease
BBB: Blood-brain barrier
CBD: Cannabidiol
CSF: Cerebrospinal fluid
DAMPs: Damage-associated molecular patterns
DON: 6-Diazo-5-oxo-norleucine
EAAT-2: Excitatory amino acid transporter 2
i.c.v.: Intracerebroventricular
i.p.: Intraperitoneally
HIV: Human immunodeficiency virus
IL-1β: Interleukin-1β
PAMPs: Pathogen-associated molecular patterns
PD: Parkinson’s disease
PP-2: Pyrazolopyrimidine-2
RA: Rheumatoid arthritis
T2DM: Type 2 diabetes mellitus
TBI: Traumatic brain injury
THC: Tetrahydrocannabinol
TLR: Toll-like receptor
TNF: Tumor necrosis factor
